# Open partial or transoral laryngectomy – total laryngectomy today

**DOI:** 10.3389/fonc.2025.1520524

**Published:** 2025-08-15

**Authors:** Dietmar Thurnher, Ricard Simo, Giovanni Succo, Isabel Vilaseca, Christian Simon

**Affiliations:** ^1^ Department of Otorhinolaryngology, Medical University of Graz, Graz, Austria; ^2^ Department of Otolaryngology, Guy’s and St Thomas’ NHS Foundation Trust, London, United Kingdom; ^3^ ENT Clinic - Head and Neck Cancer Unit, San Giovanni Bosco Hospital, Turin, Italy; ^4^ Otolaryngology and Head and Neck Surgery Unit, Hospital Clinic, Barcelona, Spain; ^5^ Department of Otolaryngology-Head and Neck Surgery, CHUV, University of Lausanne, Lausanne, Switzerland

**Keywords:** total laryngectomy, partial horizontal laryngectomy, transoral laser surgery, organ preservation, voice rehabilitation

## Abstract

Total laryngectomy, a surgical procedure involving the complete removal of the larynx, has been a crucial treatment for advanced laryngeal cancer since its introduction in 1873. Over the past 150 years, this procedure has evolved significantly, with improvements in surgical techniques, postoperative care, and rehabilitation methods leading to better survival rates and quality of life for patients. While organ-preserving approaches like radiochemotherapy have gained prominence in recent decades, total laryngectomy remains an essential option for cases of advanced cancer or when other treatments fail. This review explores the history, development, and current role of total laryngectomy in treating laryngeal cancer, as well as comparing it to alternative surgical approaches like open partial laryngectomy and transoral laser microsurgery or organ preservation protocols.

## History of total laryngectomy

The pioneering first total laryngectomy in a human patient was performed by the German surgeon Theodor Billroth in Vienna on December 31, 1873 (1), marking a significant milestone in treating advanced laryngeal cancer (2). Initially associated with high mortality rates of around 50% due to complications like pneumonia and sepsis, the procedure saw rapid improvements. Themistocles Gluck introduced a two-stage approach in 1881 to reduce risks, while Johannes Sørensen refined it into a single-stage operation by 1890, significantly lowering mortality rates. By the early 20th century, advancements in surgical techniques and postoperative care had dramatically improved outcomes, with mortality rates dropping from 44% to 8.5% between 1889 and 1900 (3).

Throughout the 20th century, surgical techniques for total laryngectomy underwent significant refinements. The introduction of neck dissection as a routine part of the procedure by George Washington Crile improved outcomes by addressing lymphatic spread. In the 1950s, Martin and Ogura standardized the combination of total laryngectomy with neck dissection, establishing a comprehensive approach to treating advanced laryngeal cancer (1).

The introduction of tracheoesophageal puncture by Singer and Blom in 1980 revolutionized voice restoration post-surgery, enhancing patients’ quality of life (4).

These advancements, coupled with improvements in anesthesia and postoperative care, have dramatically reduced complication rates and improved survival outcomes over the last 150 years (5).

Surgical approaches for treating laryngeal cancer have evolved significantly, offering various options depending on the stage and location of the tumor, including open partial laryngectomy, transoral laser resection and, as a recent development, transoral robotic surgery.

## Survival after total laryngectomy today

Survival rates following total laryngectomy vary significantly based on the primary tumor location within the larynx. Glottic cancers demonstrate the highest 5-year overall survival rate at approximately 70%, attributed to earlier detection due to noticeable voice changes. In contrast, subglottic tumors have a lower survival rate of around 40%, while retrocricoid tumors show the poorest prognosis with only a 15% 5-year overall survival rate (6). These disparities highlight the importance of tumor location in predicting outcomes and underscore the need for tailored treatment approaches and follow-up strategies for different laryngeal subsites. Tumor stage and comorbidities significantly impact survival outcomes after total laryngectomy. Advanced T-stage, particularly T4, is associated with poorer prognosis, while patients with T3 tumors generally have better survival rates (7). The presence of severe comorbidities, as measured by the ACE-27 scale, is a strong predictor of reduced overall survival and patients with no more than two comorbidities and absence of cardiovascular comorbidities at cancer diagnosis have better survival prospects (8).

## Survival outcomes for total laryngectomy vs. radiochemotherapy?

Total laryngectomy and radiochemotherapy are two primary treatment options for advanced laryngeal cancer, each with significant implications for patient survival and quality of life. While both approaches aim to control the disease, they differ substantially in their impact on speech, swallowing, and overall functioning. Long-term survival rates are comparable between the two treatments, but quality of life outcomes can vary considerably, particularly in areas such as communication, eating, and social interactions.

Recent literature comparing survival outcomes between radiochemotherapy and total laryngectomy for advanced laryngeal cancer shows mixed results, with some studies favoring surgical approaches. However, a 2023 meta-analysis of T3 laryngeal cancers found no statistically significant difference in 2-year, 3-year, and 5-year overall survival (OS) rates between total laryngectomy and concurrent chemoradiation, demonstrating 5-year OS rates of 54.2% for TL and 52.7% for radiochemotherapy, respectively and, both TL and CRT showed significantly better OS outcomes compared to radiation therapy alone, which was 40,8% (9). These findings suggest that while TL and radiochemotherapy offer comparable survival outcomes for advanced laryngeal cancers, both are superior to RT alone. However, organ preservation remains an important consideration. For example, the DeLOS-II trial reported superior OS and tumor-specific survival for patients undergoing larynx preservation protocols compared to TL followed by adjuvant therapy (10). On the other hand, just recently Pfuetzenreiter et al. showed that patients with advanced laryngeal cancers have better surviving rates when treated by total laryngectomy as a first treatment option instead of radiochemotherapy. In their study patients with T3 cancers had similar survival rates with both treatment options. Finally, feeding tube dependency was more likely with organ preservation protocols (11).

Taken together, the choice between total laryngectomy and radiochemotherapy should consider factors such as patient fitness, tumor characteristics, and the potential for organ preservation.

## Open partial horizontal laryngectomy

Vertical partial laryngectomy (VPL) and horizontal partial laryngectomy (OPHL) represent two distinct surgical approaches, each modifying the pharyngo-laryngeal anatomy in unique ways. VPL involves a vertical section of the thyroid cartilage, while HPL utilizes a horizontal approach. Both techniques can be employed for similar indications, including cT1-cT3 anterior laryngeal cancers (12), however since the advent of transoral laser surgery for the treatment of glottic cancer vertical partial laryngectomy techniques are used less frequently.

The European Laryngological Society has classified OPHL into categories such as supraglottic, supracricoid, and supratracheal laryngectomy (13), while VPL encompasses various techniques proposed by different surgeons.

The types of OPHL are based on the extent of the resection:

Type I: (Supraglottic Laryngectomy): Removes structures above the vocal cords while preserving them, used for supraglottic tumors.

Type II (Supracricoid Laryngectomy): Resects glottic and supraglottic structures but preserves the cricoid cartilage. Variants include cricohyoidopexy (CHP) and cricohyoidoepiglottopexy (CHEP).

Type III (Supratracheal Laryngectomy): Extends resection below the cricoid cartilage for more extensive tumors. This can be expanded to include adjacent structures like an arytenoid or part of the tongue base if needed.

## Survival outcomes for partial laryngectomy

Partial laryngectomy techniques like Type II (supracricoid partial laryngectomy) offer significant advantages over total laryngectomy in terms of functional outcomes and quality of life, while maintaining comparable oncological control for appropriately SELECTED patients. Furthermore, supracricoid partial laryngectomy achieved similar 5-year overall survival (83%) and disease-free survival (76.3%) rates compared to total laryngectomy for intermediate and select advanced stage laryngeal cancers, as demonstrated by Crosetti et al (14). Overall, patients undergoing SCPL report better global health status, quality of life, and general activities scores compared to TL (15) patients with preserved lung-powered speech and swallowing function without a permanent stoma, unlike obviously with total laryngectomy. Of importance, these data are limited to patients younger than 70 years with adequate neurologic and cardio-pulmonary function. The trend of minimal invasive surgery lead to described a lateral cervical approach in order to perform neck dissection and open laryngectomy whilst respecting the oncological radicality and preserving the surrounding healthy tissues at the same time (16, 17).

## History of transoral laser surgery

Transoral laser microsurgery (TOLMS) has revolutionized the treatment of laryngeal cancer since its introduction in 1972, offering comparable oncological outcomes to traditional open surgeries while providing better functional results and quality of life for patients. As reported by multiple studies, TOLMS and open partial laryngectomy (OPL) show similar overall survival rates for early-stage laryngeal cancer, but TOLMS is associated with shorter hospital stays, faster recovery, and higher rates of laryngeal preservation. The roots of transoral laser microsurgery can be traced back to 1852 when Horace Green performed one of the earliest transoral procedures using a bent tongue spatula and sunlight. Significant advancements followed, including Bernhard Fraenkel’s first transoral resection of laryngeal cancer in 1886 and Kirstein’s development of specialized endoscopes in the 1890s (18). The 20th century saw crucial innovations such as suspension laryngoscopy in the 1920s and microscopic visualization of the larynx in the 1960s, paving the way for Strong and Jako’s groundbreaking introduction of TOLMS using the carbon dioxide laser in 1972 (19). During the 1980s and 1990s, the German Otorhinolaryngologist Wolfgang Steiner significantly advanced TOLMS techniques in Europe while adoption lagged in the United States. He introduced the concept of tumor transection *in situ*, challenging the traditional en-bloc resection approach (20) thereby demonstrating that piecemeal tumor removal could achieve better deep-margin clearance under microscopic guidance. Over time Steiner expanded TOLMS applications to larger tumors and more advanced stages of laryngeal cancer. In 2000 and in revised form in 2007 the European Laryngological Society formulated a classification system for endoscopic chordectomies (21), which includes VI main types with subclassifications: Type I or Subepithelial cordectomy, Type II or Subligamental cordectomy, Type III or Transmuscular cordectomy, Type IV or total cordectomy, Type V or Extended cordectomy (Va: includes the anterior commissure and the contralateral cord; Vb: includes the arytenoid; Vc: includes the subglottis and 5d: includes the ventricle) and Type VI for the anterior commisur.

## Survival outcomes for TOLMS

Studies have shown that CO2 laser TOLMS achieves high rates of local control (90-94%) and low rates of salvage total laryngectomy (0-4%) in early-stage glottic cancers (22, 23).

The technique’s versatility has expanded its application to more advanced laryngeal tumors, including selected T2-T3 cases, while maintaining favorable oncological and functional outcomes (24).

## Survival- and functional outcomes of TOLMS compared to OPL

Both TOLMS and OPL provide similar oncological outcomes in terms of overall survival and disease-specific survival rates. Studies have shown that there is no significant difference in these survival rates between the two approaches for early-stage laryngeal cancer (25). Regarding local control rates there seems to be a higher rate for OPL in specific cases, such as tumors with deeper pathological infiltration or even vocal fold impairment (26). Of importance, these data are limited to patients younger than 70 years with adequate neurologic and cardio-pulmonary function. Regarding functional outcomes, TOLMS is associated with shorter hospitalization times and quicker recovery compared to OPL. Patients undergoing TOLMS typically experience less postoperative pain, fewer complications, and a faster transition to oral feeding (27). Thus TOLMS’ minimally invasive approach makes it an excellent indication for older patients who cannot undergo external partial surgery or chemoradiotherapy (28).

## Robotic-assisted partial and total laryngectomy

The use of robotic technology in laryngeal surgery has been of interest for its potential to perform less invasive procedures and reduce patient mortality. A pivotal study by the GETTEC, published in 2018, involved a prospective cohort of 122 patients, primarily with T1 and T2 laryngeal cancers, who underwent transoral robotic supraglottic laryngectomy. The study reported a positive margin rate of 6.6% and a close margin rate (between 1mm and <3mm) of 41.8%. Unfortunately, three patients passed away due to surgery-related complications. The loco-regional control rates were 91.8% and 87.7% at 2 and 5 years, respectively (29).

A 2020 literature review pooled data from 422 patients, most with T1 and T2 supraglottic cancers, who underwent robotic supraglottic resections. Nearly half of these resections were classified as ELS type IV. The study noted significant variability in the time it took for patients to resume swallowing, ranging from day 1 to six weeks post-surgery, highlighting the challenge of establishing consistent endpoints for swallowing recovery (30). An analysis of 500 patients across studies found an overall bleeding rate of approximately 3.75% (31). More extensive robotic-assisted procedures, such as supra-cricoid partial laryngectomies, have demonstrated similar outcomes to open surgeries (32).

Robotic cordectomies have also been explored, with one review covering 114 patients. In 4% of these cases, conversion to TOLMS was required. The positive margin rate was 4.5%, and local control at 2–5 years was an impressive 89.3%. However, 15.8% of patients required nasogastric tubes, 22.3% needed a tracheostomy, and the overall complication rate reached 12.6% (33).

Finally, robotic-assisted total laryngectomies have been documented in 29 cases. Four of these surgeries required conversion to open procedures. Of the 25 patients who underwent the robotic approach, five developed fistulas, and two experienced postoperative bleeding. Notably, 14 patients had previously received chemotherapy or radiation therapy (34).

In conclusion, transoral robotic partial laryngectomies offer promising outcomes with manageable complication rates comparable to those seen with TOLMS, particularly in treating T1 and T2 cancers. Robotic-assisted cordectomies seem feasible but may inflict substantial comorbidities. Robotic-assisted approaches for more advanced laryngeal tumors are less common. In particular robotic total laryngectomies, while technically feasible, remain rare and underreported. Further studies are necessary to better evaluate the long-term benefits and broader applicability of robotic-assisted total laryngectomy.

## Conclusion

Total laryngectomy remains an important treatment option for advanced laryngeal cancers today, though its role has evolved in the era of organ preservation.

It is now primarily reserved for T4a tumors with cartilage invasion, cases where non-surgical treatments have failed, or when laryngeal function is already severely compromised. Minimally invasive approaches like transoral laser surgery using the microscope or an exoscope or transoral robotic surgery total laryngectomy (TORS-TL) are being explored, potentially offering reduced scarring and faster recovery for select patients. These advancements seek to address the significant quality of life impacts associated with total laryngectomy while maintaining its role as an effective treatment for advanced laryngeal cancers.
